# Genetic connection between cell-wall composition and grain yield via parallel QTL analysis in *indica* and *japonica* subspecies

**DOI:** 10.1038/s41598-017-12903-5

**Published:** 2017-10-02

**Authors:** Zuopeng Xu, Shance Li, Changquan Zhang, Baocai Zhang, Kongzhi Zhu, Yihua Zhou, Qiaoquan Liu

**Affiliations:** 1 Jiangsu Key Laboratory of Crop Genetics and Physiology/Key Laboratory of the Ministry of Education for Plant Functional Genomics, College of Agriculture, Yangzhou University, Yangzhou, 225009 China; 2 State Key Laboratory of Plant Genomics, Institute of Genetics and Developmental Biology, Chinese Academy of Sciences, Beijing, 100101 China; 3 Co-Innovation Center for Modern Production Technology of Grain Crops of Jiangsu Province/Joint International Research Laboratory of Agriculture and Agri-Product Safety of the Ministry of Education, Yangzhou, 225009 China; 4 University of Chinese Academy of Sciences, Beijing, 100049 China

## Abstract

Grain yield is a complicated trait, which is highly associated with biomass productivity. The cell wall is a central element of biomass, and its biogenesis contributes to plant architecture and development. However, the genetic link between cell-wall property and grain yield is largely unclear. Here, we report on identification of quantitative trait loci (QTLs) for grain yield-related traits and cell-wall composition with a set of chromosomal segment substitution lines (CSSLs) that were generated by using 9311, an *indica* cultivar as donor, and Nipponbare, a *japonica* cultivar as recipient. Nipponbare and 9311 showed significant differences in grain yield-related traits and cell-wall composition. Genotyping with molecular markers, 125 lines covering 95.6% of the whole genome of 9311 were employed for phenotypic and chemical examinations. Thirty-seven QTLs for grain yield-related traits and nineteen QTLs for cell-wall composition have been identified. In addition to correlation analysis, we found overlapped and closely linked QTLs for two sets of traits. Fine-mapping further narrowed a QTL for cellulose content together with *HD17*, a known QTL for heading date and grain yield, suggesting that plants may regulate cell wall biogenesis and grain yield via related means. Our study provided genetic clues for cloning QTLs for both complicated traits.

## Introduction

Cell walls, consisting of a complex polysaccharide network, encase plant cells and are characteristic plant cellular structures. These structures are enriched in cellulose, hemicelluloses and pectins, which represent the most abundant natural biopolymers on Earth^1^. Without cell walls, plant cells loose their shape, indicating fundamental roles on plant morphogenesis and development. Land plants generally possess at least 35 types of cells that are surrounded by different wall structure and properties compatible with their functions in various tissues and across developing stages.

It has been estimated that cell wall synthesis and integration involve more than 1000 genes^2^. Based on characterization of mutants that showed defects in plant growth and mechanical strength, multiple glycosyltransferases (GTs), glycosyl hydrolases (GHs), and other proteins have been found implicated in wall synthesis. It is well known that cellulose synthesis is catalyzed by cellulose synthase subunits belonging to GT2 family at the plasma membrane^3,4^. Non-cellulosic polysaccharides are synthesized by multiple cellulose synthase-like proteins (CSLs) and GTs, including GT8, GT43, GT47, and GT61 family members, etc^5–10^. Korrigan (KOR), a GH9 member, and Chitinases1 (CTL1), a GH18/19 protein, are also required for cellulose biosynthesis^11,12^. Besides GT and GH, other proteins, such as COBRA, affect cellulose synthesis and deposition in Arabidopsis and rice^13,14^. Enzymes involved in substrate synthesis and transportation are also needed for cell-wall biosynthesis^15,16^. Two classes of proteins, xyloglucan endotransglycosylases (XET) and expansins, modify the cell wall and integrate new polysaccharides during cell expansion^17,18^. As some components and newly synthesized polysaccharides need to be secreted to the plasma membrane and apoplast, proteins related to cytoskeleton dynamics and vesicle trafficking are required^19–22^. In addition to cell-wall metabolic processes, transcriptional regulation constitutes another level to modulate cell-wall biosynthesis^23–25^. Upstream of the transcriptional regulatory network, receptor-like kinases acting as sensors of internal and external stimuli are responsible for perception and transduction of signals to attune downstream processes^26,27^. Therefore, control of cell wall biogenesis requires the involvement of multiple metabolic and regulatory processes. Cell-wall composition is indeed a highly complex trait that possibly can be dissected as quantitative trait loci (QTL). QTLs for cell-wall properties, such as cell-wall digestibility, lignin content, and sugar composition, have been characterized in several plant species^28–30^. *CSLF6* is the first cloned cell-wall QTL^6^. As characterization of cell-wall properties is laborious, the wall-related genes identified by QTL mapping are very limited.

Nevertheless, QTL cloning is a powerful approach for identifying major genes for agronomic complex traits, such as grain yield. Rice (*Oryza sativa* L.) is a major staple food crop feeding about three billion people, and the most important trait in rice production is grain yield. *Gn1a*, a major QTL controlling grain number, was cloned based on natural allelic variations^31^. *DEP1*, the locus that governs the panicle architecture and grain number was isolated by QTL positional cloning^32^. Grain size and grain weight are also key determinants for grain yield. Till now, some QTLs for grain size (GS) and grain weight (GW) control have been cloned. For example, *GS3* encodes a transmembrane protein, *GW2* encodes an E3 ligase, *GW8* encodes SPL16, and *Grain length3.1* (*GL3.1*) is a phosphatase, suggesting that cell-cycle or cell-size regulators are required for grain yield control^33–36^. Plant height (PH) and heading date (HD) are other determinants of grain yield. The most famous gene for plant height control is *sd1*, which greatly enhanced grain productivity by improving lodging resistance in the 1960s^37^. But the first cloned QTLs for plant height and heading date are *PH1* and *HD1* ^38,39^, respectively. Moreover, these agronomic traits are often correlated. For example, *GS2*/*GL2* that controls grain size also affects grain weight^40,41^. *Ghd7*, a QTL for HD, controls plant height and grain number^42,43^. In addition to these major loci, numerous minor loci also contribute to grain yield, which might be more useful in genetic breeding. Due to the small effects on genetic variability, the connection between genotype and phenotype is hard to bridge, which impedes cloning of the minor QTLs.

Metabolites that act as either causes or effects of complex traits may build a bridge between genotype and phenotype^44^. As the technical advances progress on high throughput examination of metabolites, comprehensive metabolic profiling and large-scale gene characterization by association mapping have been achieved^45,46^. Discovery of genetic links between metabolic and phenotypic variation becomes implementable. Correlation analysis of fruit metabolic loci and morphological genes for yield-associated traits in tomato introgression lines has revealed the links between nutritional metabolites and organoleptic traits^47^. By using the same approach, lignin precursors identified by genome wide association study (GWAS) are correlated with plant height^45^. A major locus controlling the trigonelline level identified by metabolic GWAS in 489 rice varieties is identical to a QTL for grain width^48^. Recently, two loci (*Lin5* and *SSC11.1*) regulating sugar content and tomato flavor were found negatively correlated to fruit size^49^. Therefore, discovery of the metabotype-phenotype linkage is a useful approach for interactive functional genomics, especially for cloning minor QTLs for complex traits.

Cell wall is the central element of biomass. It is well known that extended heading date and increased plant height lead to more biomass and consequently promote grain yields. Hence, cell wall synthesis may be a valuable metabolic marker associated with grain yields. However, genetic links between cell-wall composition and grain yield-related traits are unclear.

Here we report on generation of a chromosome segment substitution lines (CSSL) by using Nipponbare (NP), a representative cultivar for *japonica*, as recipient parent and 9311, a representative cultivar for *indica*, as donor parent. Based on the variations between the two rice subspecies *indica* and *japonica*, parallel studies on six grain yield-related traits and cell-wall composition were performed in the CSSLs, and the corresponded QTLs for these traits were mapped. The overlapped QTLs for the two sets of traits have been identified. Fine-mapping of a QTL for cellulose content provided further evidence for the genetic connection between both sets of traits. Our study thus sheds lights on dissecting the machinery on cell-wall biosynthesis and grain-yield improvement in rice.

## Results

### Analysis of phenotypic variations between NP and 9311

**Figure 1:**
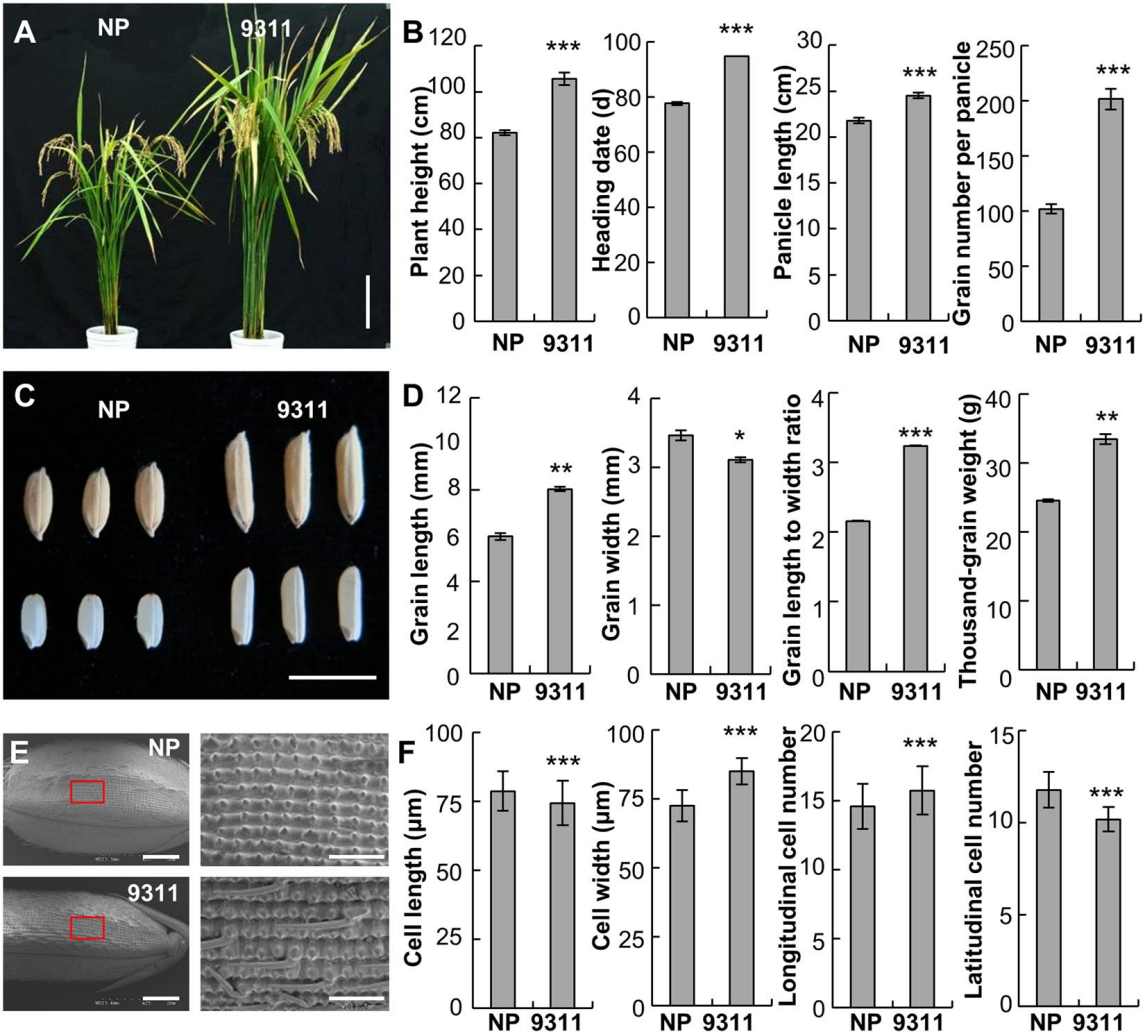
Phenotypic difference between NP and 9311. (**A**) The mature plants of NP and 9311. (**B**) Quantification of the morphological phenotypes in NP and 9311. Error bars represent the mean value ± SD (n ≥ 9 plants). (**C**) Grain shape of NP and 9311. (**D**) Measurement of the grain size and grain weight of NP and 9311. Error bars represent the mean value ± SD (n ≥ 50 grains harvested from at least 10 plants). Error bars of thousand-grain weight is the mean value ± SD (n = 3 replicates). (**E**) Scanning electron microscopic photographs of the outer surface of a glume. The right panels are the enlarged view circled by the rectangles. (**F**) Quantification of cell length, cell width, and cell number at the longitudinal and latitudinal direction in glume. Error bars represent the mean value ± SD (n ≥ 500 cells from 12 seeds). ***P < 0.001, **P < 0.01, and *P < 0.05 by two-tailed *t*-test. Bar = 15 cm in (**A**), 1 cm in (**C**), and 1 mm for left panel and 250 μm for right panel in (**E**).

Among the morphological and physiological differences between NP and 9311, biomass abundance is the fundamental one. *Indica* variety 9311 generally has more biomass than *japonica* variety NP, which is in agreement with that 9311 has increased heading date, indicating as a longer vegetative growth period, increased plant height, larger panicles, and more grains than NP (Fig. 1A and B). Another morphological difference between 9311 and NP is the grain shape. Variety 9311 has long and slender grains whereas those of NP are short and wide (Fig. 1C and D). Hence, the ratio of grain length to width is significantly increased in 9311, resulting in increased thousand-grain weight compared to NP (Fig. 1D). To address this difference at a cellular level, we investigated cell shape on glumes by scanning electronic microscopy. Surprisingly, cells in 9311 glumes were shorter in length but wider than those in NP (Fig. 1E and F). Quantification of cell numbers in glumes showed that 9311 had more cells at the longitudinal direction, but fewer cells at the latitudinal direction (Fig. 1F). Therefore, all these differences are likely derived from varied ability of biomass productivity between NP and 9311.

### NP and 9311 have different cell-wall composition

**Figure 2:**
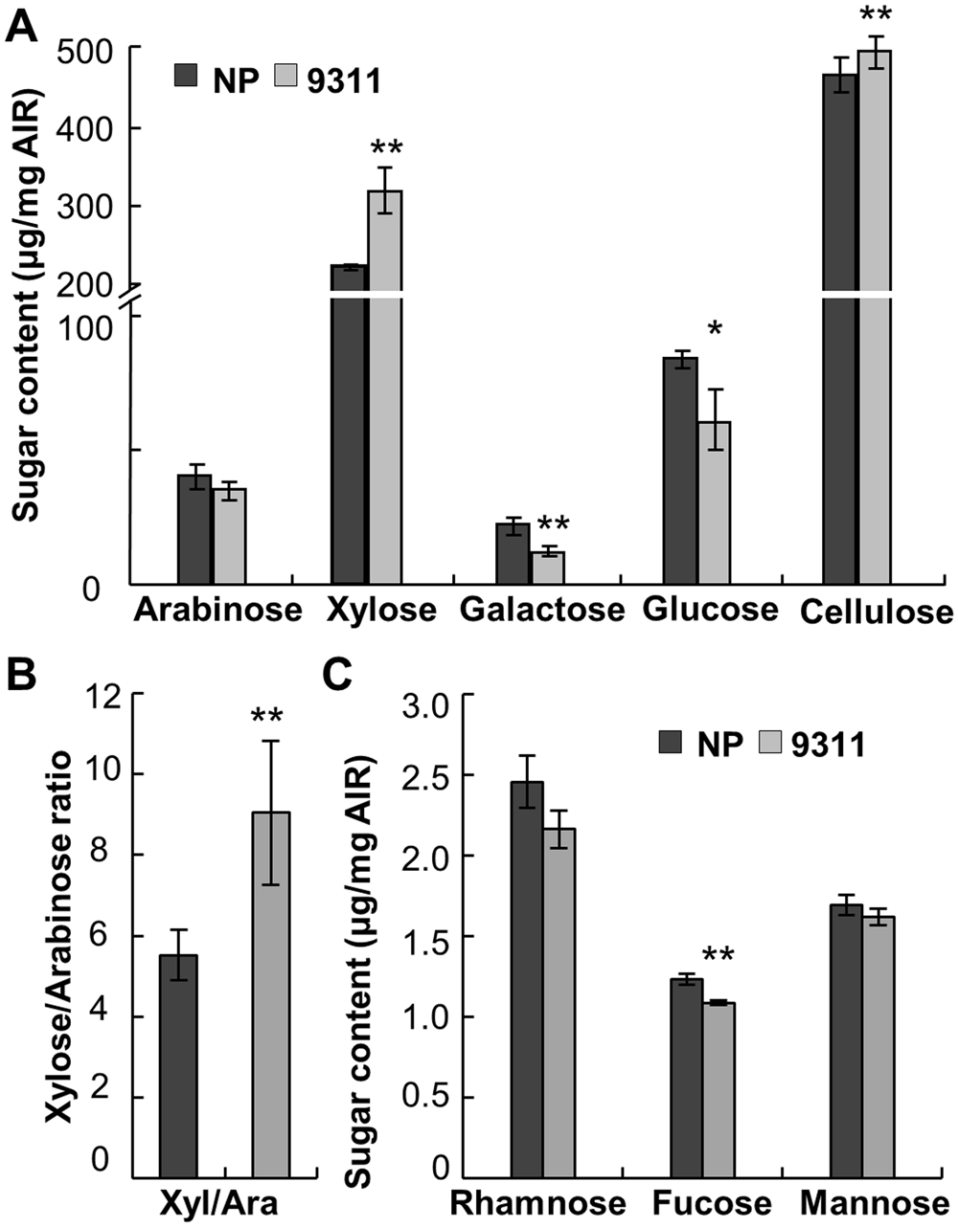
Measurement of the cell-wall composition in NP and 9311. (**A**) The content of major neutral sugars and crystalline cellulose in wall residues from internodes. (**B**) The ratio of xylose and arabinose content. (**C**) The content of minor neutral sugars in wall residues from internodes. Error bars represent the means ± SD (*n* = 4 replicates using AIR from 10 mature plants. **P < 0.01 and *P < 0.05 by two-tailed *t*-test.

As cell walls represent the major element of plant biomass, we investigated whether variations on cell-wall composition exist in the two varieties. Because internodes constitute a central source of rice biomass and contribute directly to plant height and grain traits by linking source and sink, internodes were subjected to cell-wall composition examination. The content of cellulose and xylose, the two major components of cell wall, was significantly increased in 9311 (Fig. 2A). In contrast, galactose and glucose that are often present in hemicellulose or amorphous cellulose were reduced in 9311 (Fig. 2A). As arabinosyl residues (Ara) present as side chains on xylans in rice, the ratio of xylose to arabinose (XA) reflects the xylan substitution level. Although the arabinose content was not significantly changed, more xylose content caused higher XA ratios in 9311 (Fig. 2B), indicating that 9311 has lower arabinosyl substitution on xylan compared to NP. Due to a very low level of pectin presenting in rice cell walls, the pectic sugars, rhamnose and fucose, were hard to be detected (Fig. 2C). Therefore, NP and 9311 have different cell-wall composition in the internodes.

### Construction of CSSLs

To identify QTL for cell-wall composition and grain yield, we constructed a CSSL population by backcrossing the previously reported 57 CSSLs^50^ with NP and followed by self-crossing. With the aid of molecular marker-assisted selection, a total of 351 CSSLs were obtained. These CSSLs were further genotyped by 357 polymorphic molecular markers that were evenly distributed across the 12 chromosomes. Subsequently, 138 core lines that may cover the whole 9311 genome were selected and subjected to re-sequencing.

Consequently 125 lines that had convincing re-sequencing data were used to construct a graphical genotypic map. As shown in Supplemental Fig. 1, each CSSL contains one to seven substituted segments. In more detail, 53 lines harbored only one substituted segment, 44 lines contained two introgressed segments, and 28 lines had more than two segments (Supplemental Fig. 1). The total length of the 353 substituted segments was around 1999.76 Mb, which is five times of the 9311 genome. Re-sequencing analysis revealed that the length of individual substituted segments ranged from 0.02 to 24.6 Mb, with an average length of 5.6 Mb. Bin mapping showed that five chromosomes, Chr. 2, 3, 6, 7 and 9, were fully covered by the substituted segments, whereas the remaining chromosomes had 81.5% to 98.5% coverage. The uncovered regions, shown as seven small gaps, are about 4.4% of the whole 9311 genome. Therefore, this CSSL population covers ~95.6% of the 9311 genome, which is suitable for genome-wide surveys.

### The CSSLs display a wide range of variation in grain yield-related traits and cell-wall composition

We investigated six grain yield-related traits, including PH, HD, and grain size/weight in the CSSLs and the two parents. PH, HD, and grain length to width ratio (GLWR) were continuous within the range of two parents, and a few lines were superior to their parents (Supplemental Fig. 2). However, for GL, grain width (GWD), and thousand-grain weight (TGW), more lines were superior to their parents, indicating that loci-interacting effects might exist in these traits (Supplemental Fig. 2). The wide range of phenotypic variation suggested complexity in these agronomic traits.

We also analyzed cell-wall composition of the mature culms from the CSSLs. To guarantee accuracy of examination, we performed variation analysis on the content of four major sugars and cellulose, as that of rhamnose, fucose and mannose is too low to be precisely measured. As shown in Supplemental Fig. 3, the content of the four sugars and cellulose showed a continuous distribution among the CSSLs with transgressive segregation on both sides. The ratio of xylose to arabinose, reflecting arabinoxylan structure, was also continuously distributed. Therefore, cell-wall composition belongs to a complex trait.

### Cell-wall composition is correlated to grain yield-related traits

To determine whether cell-wall composition is correlated with the grain yield-related traits, we performed correlation analysis. The content of cellulose and four sugars and XA ratio showed varied correlation coefficient against six grain yield-related traits (Table 1). PH, one of the most important traits for crop architecture and grain yield, was significantly associated with cellulose content and XA ratio. Similarly, HD that indicates vegetative growth period was associated with XA ratio and the content of cellulose, xylose and galactose (Table 1), demonstrating that biosynthesis of cellulose and xylan is correlated with PH and HD. GWD, GL and TGW are direct determinants of grain yields. Interestingly, XA ratio displayed a high correlation with TGW and GL, while the glucose and galactose content were significantly correlated with TGW and GWD (Table 1). The highest correlation coefficient between cell-wall composition (XA ratio) and grain yield-related traits (TGW) was 0.44, indicating that xylan structure may contribute to TGW and grain yield. As positive controls, 0.85 or 0.6 correlation coefficients were found between the abundance of arabinose and galactose or xylose due to the existence of arabinogalactan and arabinoxylan in rice cell walls. Similarly, around 0.6 correlation coefficients were showed between PH and HD and between GL and TGW (Table 1), which is in agreement with previous reports^51^.

**Table 1:** Correlation coefficient between cell wall components and grain yield-related traits.

Traits	Ara	Xyl	Gal	Glc	XA	Cel	PH	HD	GL	GWD	GLWR	TGW
Ara	1											
Xyl	0.60^**^	1										
Gal	0.85^**^	0.17	1									
Glc	0.66^**^	0.15	0.75^**^	1								
XA	−0.49^**^	0.39^**^	−0.80^**^	−0.57^**^	1							
Cel	0.01	0.20^*^	−0.15	−0.13	0.28^*^	1						
PH	−0.05	0.14	−0.20^*^	−0.18^*^	0.23^*^	0.34^**^	1					
HD	−0.08	0.23^*^	−0.31^**^	−0.12	0.34^**^	0.21^**^	0.62^**^	1				
GL	−0.08	0.16	−0.18^*^	−0.19^*^	0.27^**^	0.08	0.33^**^	0.28^**^	1			
GWD	−0.22^*^	−0.1	−0.22^**^	−0.28^**^	0.18	0.13	0.22^**^	0.16	0.42^**^	1		
GLWR	0.15	0.23^*^	0.08	0.11	0.06	−0.07	0.07	0.12	0.45^**^	−0.61^**^	1	
TGW	−0.16	0.21^*^	−0.38^**^	−0.31^**^	0.44^**^	0.06	0.51^**^	0.58^**^	0.63^**^	0.42^**^	0.16	1

*P < 0.05 and **P < 0.01 by student *t* test (two tailed). Ara, Arabinose; Xyl, Xylose; Gal, Galactose; Glc, Glucose; XA, Xylose/Arabinose; Cel, Cellulose; PH, Plant height; HD, Heading date; GL, Grain length; GWD, Grain width; GLWR, Grain length-to-width ratio; TGW, Thousand-grain weight.

Taken together, the cell-wall compositions are correlated with grain yield-related traits in rice.

### QTL analysis for grain yield-related traits and cell-wall composition

QTL mapping for the grain yield-related traits and cell-wall composition was carried out based on Bin-map converted from physical map^52^. We found 56 QTLs in total. In detail, nine QTLs for PH were found located on five chromosomes with positive and negative additive effects. The contribution from 9311 indicates a positive additive effect, and that from NP is negative. The variance explained by these QTLs varied from 4.7% to 31.1%. *qPH3-1* and *qPH8* with positive effects were major loci and located on Chr. 3 and Chr. 8, whilst *qPH1-2* with negative effect was located on Chr. 1 (Table 2). We further found that *qPH1-2* and *qPH8* included the known important loci for plant height, such as *sd1* ^37^ and *Ghd8/DTH8* ^53,54^, suggesting that the mapping data are reliable. Among eleven QTLs for HD, three major loci were mapped on Chr.3 and Chr.8, which explained 17.72% to 30.81% phenotypic variance (Table 2). The 30.81% variation is likely due to the presence of *DTH8/Ghd8* ^53,54^, a major QTL for heading date, in the mapping region. The remaining QTLs had minor effects, in which *qHD6-1* included *Hd3a* ^55^ and *Hd17* ^56^, and *qHD6-2* included *Hd1* ^38^ (Table 2). For grain size or weight control, many minor QTLs with 6–11% explanation were found distributed on seven chromosomes (Table 2). Meanwhile, six major QTLs for GL, TGW, and GLWR located on Chr.2, 3 and 5 and explained 15.31% to 48.9% variance were also identified (Table 2). Within the mapping regions of *qGL3-2* and *qGWD5*, *GS3*, *GL3.2* and *GW5*, the known QTLs for grain size or weight control^36,57,58^, are located.

**Table 2:** Identification of QTLs for grain yield-related traits.

QTLs	Chr.	Bin^a^	LOD	PVE (%)	Add^b^	Position (Mb)
Plant height						
*qPH1-1*	1	Bin12	9.07	8.7	11.53	24.73–27.63
*qPH1-2*	1	Bin17	7.71	7.21	−10.5	37.95–40.68
*qPH2-1*	2	Bin20	10.05	9.82	12.25	1.1–1.3
*qPH2-2*	2	Bin52	6.61	6.05	3.72	28.32–32.53
*qPH3-1*	3	Bin74	16.81	18.72	6.55	6.92–10.56
*qPH3-2*	3	Bin97	9.18	10.19	6.24	34.98–36.40
*qPH6-1*	6	Bin140	7.15	6.61	4.19	1.31–3.77
*qPH6-2*	6	Bin173	5.26	4.7	3.5	25.89–26.83
*qPH8*	8	Bin238	24.11	31.11	7.51	4.11-4.51
Heading date						
*qHD2*	2	Bin49	7.45	1.95	1.78	26.26–27.2
*qHD3-1*	3	Bin74	23.4	8.36	2.82	6.92–10.56
*qHD3-2*	3	Bin97	36.62	17.72	10.49	34.98–36.40
*qHD5*	5	Bin130	13.45	6.87	8.17	23.29–23.38
*qHD6-1*	6	Bin140	20.92	7.11	2.6	1.31–3.77
*qHD6-2*	6	Bin161	3.4	0.83	−0.95	7.88–9.67
*qHD7*	7	Bin230	6.43	4.63	4.65	28.94–29.69
*qHD8-1*	8	Bin234	33.81	15.78	10.61	2.18–2.43
*qHD8-2*	8	Bin240	41.91	30.81	15.01	4.57–5.36
*qHD11*	11	Bin340	13.37	4.16	3.88	20.02–20.03
*qHD12*	12	Bin361	3.36	0.19	1.52	6.5–6.78
Grain length						
*qGL3-1*	3	Bin74	4.83	17.19	0.19	6.92–10.56
*qGL3-2*	3	Bin79	4.69	16.71	0.39	15.43–17.74
*qGL7-1*	7	Bin212	2.55	9.48	0.21	19.36–19.40
*qGL7-2*	7	Bin216	2.89	10.64	0.22	21.21–22.67
Grain width						
*qGWD2-1*	2	Bin35	3.88	10.89	−0.22	18.92–19.98
*qGWD2-2*	2	Bin55	2.84	7.83	−0.15	33.75–34.46
*qGWD5*	5	Bin118	2.93	8.07	−0.23	2.07–5.78
*qGWD6*	6	Bin164	2.69	7.37	−0.12	11.02–11.46
Grain length to width ratio						
*qGLWR1*	1	Bin17	2.71	10.05	0.21	37.95–40.68
*qGLWR2-1*	2	Bin18	5.6	9.36	0.12	0–1.1
*qGLWR2-2*	2	Bin35	21.06	48.89	0.27	18.92–19.98
*qGLWR3*	3	Bin79	6.45	10.97	0.1	15.43-17.74
*qGLWR5*	5	Bin118	8.61	15.31	0.19	2.07–5.78
*qGLWR6*	6	Bin169	6.69	11.44	0.12	21.55–21.82
Thousand-grain weight						
*qTGW3*	3	Bin79	4.69	16.71	0.39	15.43–17.74
*qTGW7*	7	Bin216	2.89	10.64	0.22	21.21–22.67
*qTGW8*	8	Bin234	3.69	6.36	−0.6	2.18–2.43

^a^Bin overlapping with the LOD peak of the QTL. ^b^The allelic effect is calculated as the mean effect of replacing NP alleles by 9311 alleles at the QTL. LOD, Logarithm of the odds ratio; PVE, Percentage of the trait variance explained by the QTL.

Using the same approach, a total of nineteen QTLs for the content of cellulose and four major neutral sugars and XA ratio were identified on nine chromosomes. Probably because cellulose biosynthesis is modulated by multiple genes with small effects, only one QTL for cellulose content (*qCel6*) with positive additive effect was mapped and explained 10.9% phenotypic variance (Table 3). Three QTLs for xylose content were detected, of which *qXyl7* and *qXyl10* were of negative effect and concentrated on Chr. 7 and 10, whilst *qXyl8* that had positive effect was located on Chr. 8. The variance explained by these QTLs varied from ~8% to 13.6% (Table 3). Four QTLs for the arabinose content showing positive effect were mapped on Chr. 4, 7 and 8 with around 6.4%-12.5% variance (Table 3). Similarly, four QTLs for XA ratio were targeted onto Chr. 7 and 8, in which *qXA7-1* and *qXA7-2* are major QTLs as they explained 59.2% and 17.7% phenotypic variance (Table 3). Within five QTLs for the glucose content, *qGlc1-1* and *qGlc11* explained 17.7% and 15.6% phenotypic variance (Table 3). Finally, two QTLs for the galactose content were mapped on Chr. 4 and 8, respectively (Table 3).

**Table 3:** Identification of QTLs for cell-wall composition.

Traits	QTLs	Chr.	Bin^a^	LOD	PVE (%)	Add^b^	Position (Mb)
Cellulose	*qCel6*	6	Bin140	3.11	10.90	14.29	1.31–3.77
Xylose	*qXyl7*	7	Bin188	2.60	7.97	−27.23	2.82–2.85
	*qXyl8*	8	Bin258	3.02	9.65	37.95	23.01–24.22
	*qXyl10*	10	Bin315	4.29	13.57	−49.61	14.7–19.14
Arabinose	*qAra4*	4	Bin114	2.51	6.44	4.26	33.31–33.41
	*qAra7*	7	Bin226	4.67	12.47	4.25	26.32–26.42
	*qAra8-1*	8	Bin234	3.82	10.04	3.33	2.18–2.43
	*qAra8-2*	8	Bin261	3.64	9.54	8.91	24.64–25.59
Xylose***/***Arabinose ratio	*qXA7-1*	7	Bin226	17.98	59.22	0.05	26.32–26.42
	*qXA7-2*	7	Bin230	6.76	17.75	0.03	28.94–29.69
	*qXA8-1*	8	Bin234	3.68	9.12	0.02	2.18–2.43
	*qXA8-2*	8	Bin263	3.08	7.54	0.03	25.7–26.11
Glucose	*qGlc1-1*	1	Bin8	5.29	17.72	41.60	9.44–11.05
	*qGlc1-2*	1	Bin12	2.81	6.35	18.86	24.73–27.63
	*qGlc2*	2	Bin44	2.55	5.75	8.16	22.86–24.67
	*qGlc3*	3	Bin95	3.47	7.96	12.30	31.42–34.98
	*qGlc11*	11	Bin334	4.5	15.6	15.0	18.13–18.17
Galactose	*qGal4*	4	Bin105	2.81	8.64	7.23	18.60–22.76
	*qGal8*	8	Bin234	4.40	13.96	3.35	2.18–2.43

^a^Bin overlapping with the LOD peak of the QTL. ^b^The allelic effect is calculated as the mean effect of replacing NP alleles by 9311 alleles at the QTL. LOD, Logarithm of the odds ratio; PVE, Percentage of the trait variance explained by the QTL.

By using this CSSL population, QTLs for six grain yield-related traits and cell-wall composition have been parallelly mapped on eleven rice chromosomes with the exception of Chr. 9.

### QTLs for cell-wall composition and grain yield are overlapped

Physical mapping of the above loci further showed nicely co-localized QTLs. Firstly, several grain yield-related QTLs were co-localized. QTLs for PH and GLWR overlapped on Chr. 1 (Supplemental Fig. 4). The overlapping could extend to PH and HD on Chr. 3, 6 and 8, and to HD and TGW on Chr. 8, suggesting that plant height and heading date are correlated to grain yield. Within the nineteen QTLs for cell-wall composition, QTLs for the arabinose content were co-localized with XA ratio on Chr. 7 and 8, and with the galactose and xylose content on Chr. 8 (Supplemental Fig. 5), in agreement with the existence of arabinoxylan and arabinogalactan in the rice cell-wall polymers.

**Figure 3:**
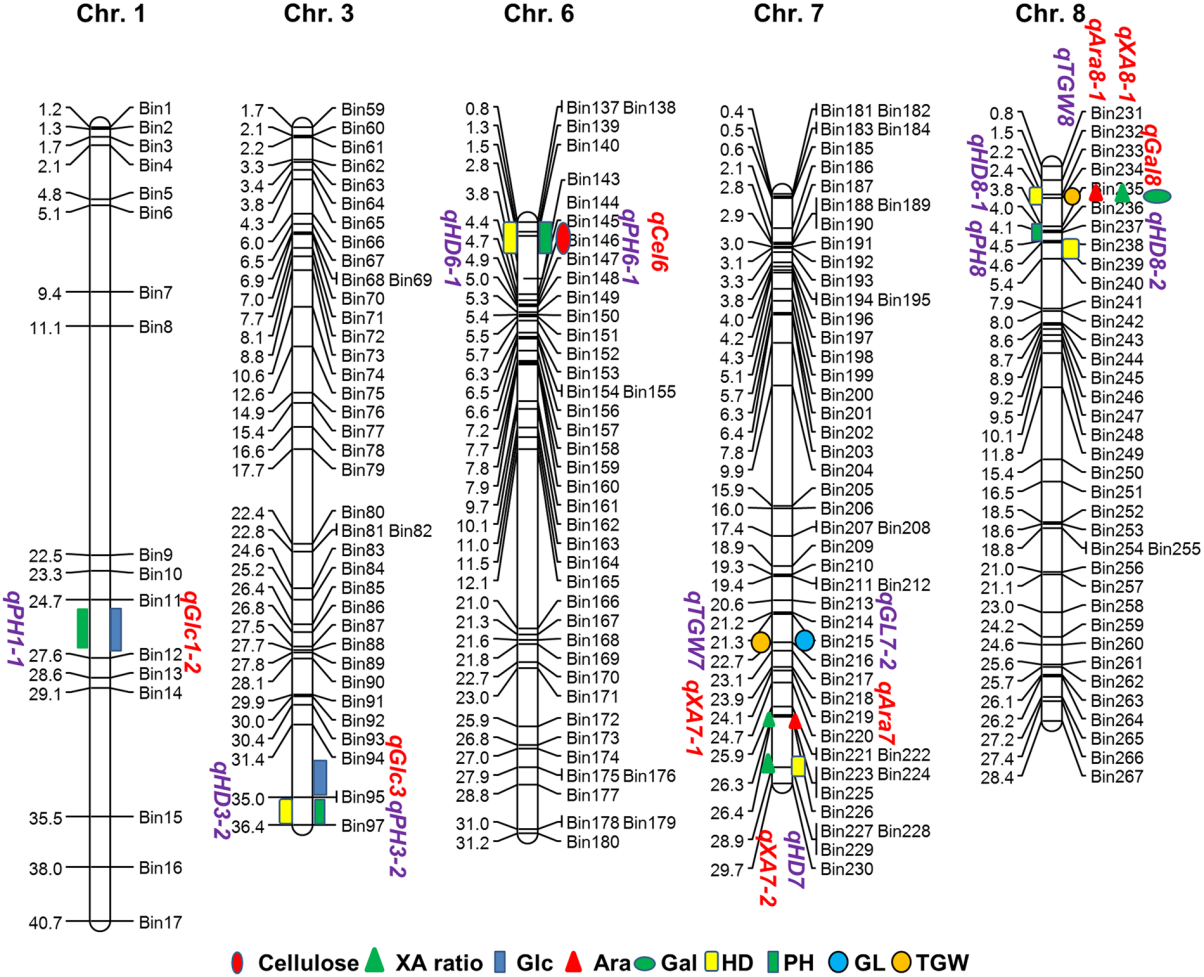
Chromosomal locations of the overlapped and adjacent QTLs for the cell-wall composition and grain yield-related traits. Different symbols indicate the positions where the corresponding QTLs are located. The numbers at the left side of chromosomes indicate the physical locations (Mb).

**Figure 4:**
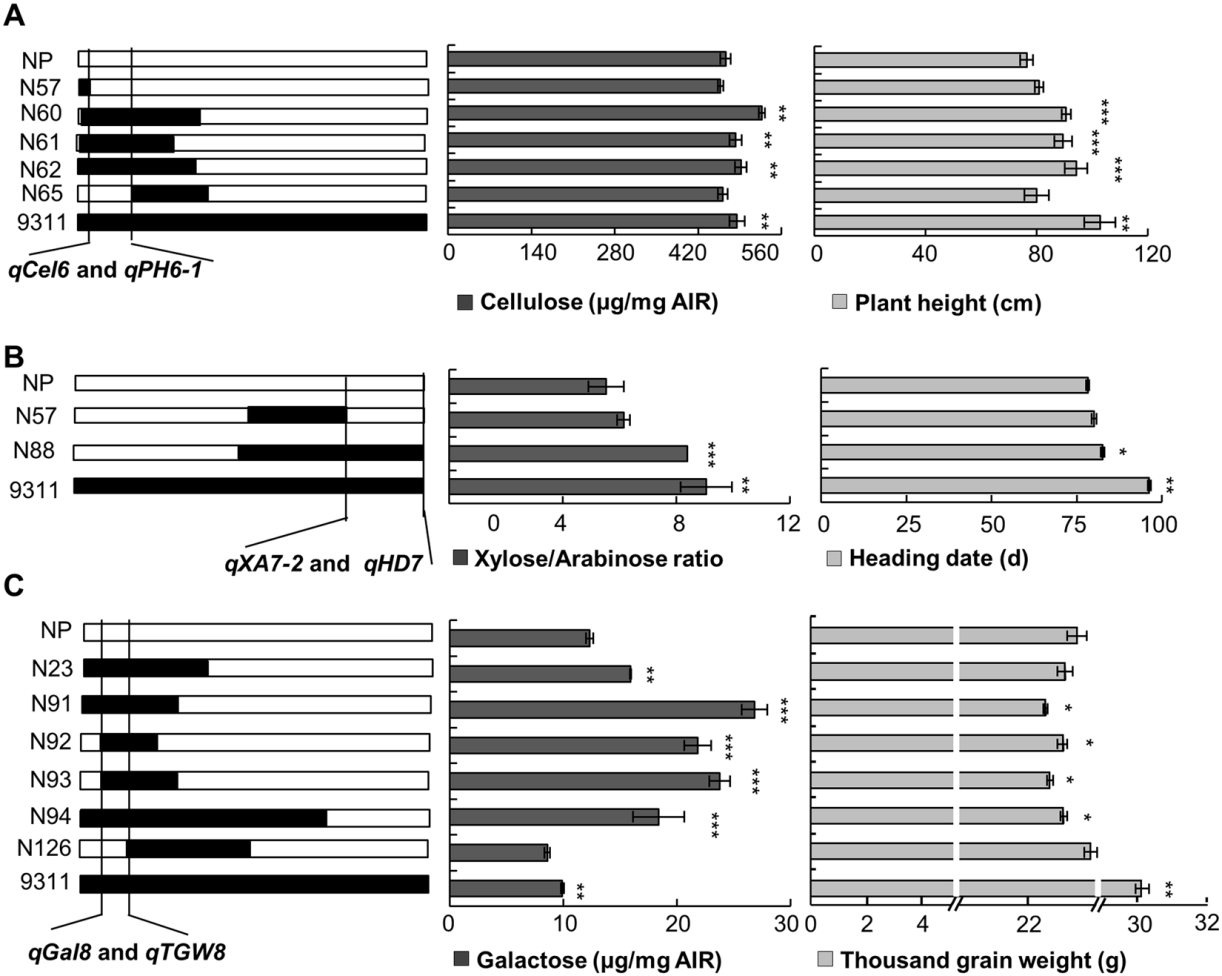
Verification of the overlapped QTLs for cell-wall composition and grain yield-related traits by the CSSLs. (**A**) The co-localized QTLs on Chr. 6 and examining the cellulose content and plant height in the CSSLs. (**B**) The co-localized QTLs on Chr. 7 and examining the XA ratio and heading date in the CSSLs. (**C**) The co-localized QTLs on Chr. 8 and examining the galactose content and thousand-grain weight in the CSSLs. The black and white rectangles in this figure represent 9311 and NP genomic region, respectively. ***P < 0.001, **P < 0.01, and *P < 0.05 by two-tailed *t*-test.

Interestingly, QTLs for the cellulose (*qCel6*), glucose (*qGlc1-2*), and galactose (*qGal8*) content, and XA ratio (*qXA7-2*) were overlapped with those for PH (*qPH6-1* and *qPH1-1*), TGW (*qTGW8*) and HD (*qHD6-1* and *qHD7*) (Fig. 3). *qAra7* and *qXA7-1* were adjacent to *qGL7* and *qTGW7*. Similar relationship was found between *qAra8-1* and *qPH8*. This finding indicated that certain genes required for cell-wall biosynthesis may affect agronomic traits. To explore evidence underlying the connection, we investigated the genotypes and phenotypes in the corresponding CSSLs. *qCel6* and *qPH6-1*/*qHD6-1* were co-targeted within a 2.46-Mb region on Chr. 6. Five substitution lines containing and not containing the 9311 fragment showed consistent tendency in cellulose content and plant height. In detail, the CSSLs possessing the 9311 fragment had increased cellulose content and plant height (Fig. 4A). *qXA7-2*, a major QTL for XA ratio, was mapped at the end of Chr. 7, where *qHD7* is located. Line 88 (N88) containing the 9311 fragment displayed higher XA ratio and longer heading date than NP and N57 that do not harbor the substitution fragment (Fig. 4B). Within the mapping region on Chr. 8, QTLs for thousand-grain weight and *qGal8* were colocated. The substitution lines harboring the 9311 fragment showed increased galactose content and slightly reduced thousand-grain weight (Fig. 4C). Taken together our data suggest that these regions are very likely to harbor QTLs synchronously controlling cell-wall biosynthesis and grain yield.

### Fine-mapping of *qCel6*

**Figure 5:**
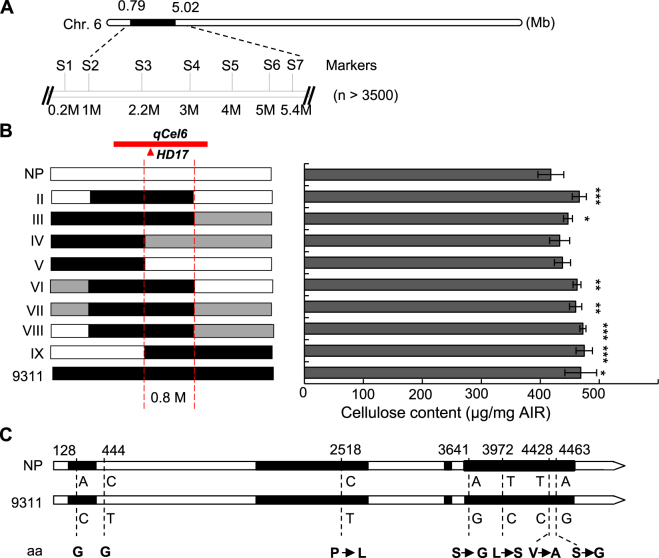
Fine-mapping of *qCel6*. (**A**) Mapping of *qCel6* in the backcross population. The numbers on chromosome 6 indicate the physical locations (Mb). S1-S7 indicate molecular markers. (**B**) Genotyping the segregation lines (left) and measuring the cellulose level in the internodes of corresponding lines (right) shown in left. n = 4. ***P < 0.001, **P < 0.01, and *P < 0.05 by two-tailed *t*-test. The black, white and grey rectangles indicate 9311, NP and heterozygous genomic region, respectively. The red bar and red dash lines indicate the QTL mapping and narrowed region of *qCel6*. The red triangle indicates the place *HD17* located. (**C**) *HD17* gene structure and allelic variations between 9311 and NP. The numbers indicate the place of SNPs located. The arrows indicate the changes of amino acid (aa).

To find a further support for this conclusion, we generated a mapping population by backcrossing N62, a line containing a 9311 fragment and contributing to high cellulose level, with NP. Based on genotyping of more than 3500 BC_8_F_2_ plants and examining the cellulose content in the internodes of plants that possess the corresponding genotypes, *qCel6* was narrowed down to a ~800 kb region, in which *HD3a*, *HD3b* and *HD17* controlling heading date and grain yields are located^55,56^ (Fig. 5A and B). Natural variations on these genes between NP and 9311 or the substitution lines were investigated by DNA sequencing. Surprisingly, we have not found any variations in the coding and promoter regions of *HD3a* and *HD3b*. Seven single nucleotide polymorphisms (SNPs) that cause five amino acid substitutions in HD17 have been identified (Fig. 5C), indicating that the increased cellulose content is likely due to variations on *HD17*. Co-segregation of *qCel6* and *HD17* in the fine-mapping assay provided further evidence for the genetic correlation between cell-wall composition and an agronomic trait.

## Discussion

CSSLs that contain one or a few donor segments in each line are widely used for QTL mapping. Since the CSSLs possess the same genetics background, QTL mapping with this population is of high accuracy and could be dissected as single Mendelian factors^59^ although preparation of CSSLs is time-consuming. Here we generated CSSLs using NP as receptor and 9311 as donor. Both parents are representative varieties for two rice subspecies and show a great genetic variance suitable for QTL association analysis. Rice is one of Earth’s major staple crops. Improvement of grain yield is the most important issue in rice production. Due to the complexity of grain yield control, a great number of genes have been inferred to be involved. However, although some major QTLs for grain size/weight have been identified^33–36,60^, the cloned loci with smaller effects are very limited. Recent works have revealed that metabotype-phenotype links are powerful to clone minor QTLs for complex phenotypic traits^44,45,47^. As cell walls are regarded as basic materials for plant growth, relevant metabolites could be annotated as causes or effects^2^. Therefore, combining QTL mapping for two sets of traits may provide a comprehensive view to understand the mechanism of grain yield control and assigning the biological functions to cell-wall synthesizing genes.

In this study, six phenotypic traits, named as grain yield-related traits, were investigated in the CSSLs. Plant height and heading date are widely studied traits and associated with crop yield^37,53^. We identified nine QTLs for PH and eleven QTLs for HD, which not only included several previously identified QTLs, such as *SD1* ^37^, *HD1* ^38^, *HD3a* ^55^, *HD17* ^56^, and *DTH8/GHD8* ^53,54^, according to the mapping region of *qPH1-2*, *qHD6-1*, *qHD6-2*, and *qHD8-1/qPH8*, but also contained several new loci, such as *qPH1-1*, *qHD3-2* and *qHD5*. Similarly, QTLs for grain size and grain weight were investigated, which identified some known QTLs. For example, *qGL3-2*/*qGLWR3* included *GS3* ^36^ and *GL3.2* ^57^, and *qGWD5* contained *GW5* ^58^. *qGL7-2* and *qGWD2-1* were adjacent to the previously reported *GL7* and *GW2*, respectively^33,61^, indicating that our mapping results are reliable. Notably, several unidentified loci were also found, such as *qGL3-1* and *qTGW7*. Although the mapping population was derived from the 57 CSSLs, three previously reported minor QTLs for grain weight^50^ have not being found here, suggesting that mapping minor loci for complex traits is of great challenge.

We parallelly analyzed cell-wall composition in the CSSLs. Consequently, nineteen QTLs for six cell-wall content characteristics were identified. Only one QTL for the cellulose content, *qCel6* that explained 10.9% of phenotypic variation, was found, indicating that cellulose property is affected by many minor loci, consistent with the notion that cellulose synthesis is very complicated and required the involvement of multiple genes^2–4^. Interestingly, *qCel6* is very likely to be an uncharacterized cell-wall synthesis related gene, because within the mapping region on Chr. 6, none of known cellulose synthesizing or regulatory genes has been reported. Arabinoxylan is the second most abundant component next to cellulose in rice cell wall. Xylose content and XA ratio are the most important parameters reflecting arabinoxylan abundance and structure. In this study, we identified three QTLs for xylose content and four QTLs for XA ratio. Previous genetic evidence demonstrated that GT43 and GT47 family members are xylan synthases^8,62,63^. However, we did not find any GT43 and GT47 members located in, or adjacent to, the xylose QTLs, suggesting that these QTLs may represent unknown genes influencing xylan biosynthesis. It is worth to point out that the mapping region of *qXyl10* includes *CESA7*, a key gene for secondary wall cellulose synthesis, in agreement with the elucidated correlation relationship between cellulose and xylan biosynthesis^8^. *OsUAM3*, encoding a putative uridine diphosphate-arabinose mutase, was adjacent to *qXA7-1*, a major QTL for XA ratio that explained up to 59.22% phenotype variation. *OsUAM3* may be a candidate for this locus. Moreover, the nineteen QTLs identified here were different from the previously reported cell-wall QTLs mapped by a CSSL population containing a common wild rice, Yuanj introgressed segment(s) in an *indica* cultivar, Teqing^30^. The distinction not only indicated the complexity of cell wall biogenesis, but also prompted us to investigate the biological functions behind these genetic clues.

Correlation analysis between different traits has been widely used to ascertain a QTL with pleiotropic functions. Several studies have reported positive correlation between PH and HD, and between GL and TGW/HD^40,41,43,51^. In this study, we found that the mapping regions for *qPH1-2* and *qGLWR1* and for *qHD8-1* and *qTGW8* were overlapped, in agreement with the previous reports that *Ghd7* and *DTH8/Ghd8* are a pleiotropic QTL, which simultaneously regulates flowering time, plant height and grain productivity^42,54^. Bridging genetic links across metabolites and phenotypic traits by paralleled QTL mapping is effective for annotation of the biological function of a metabolite, and for cloning QTLs with minor effects^44^. Through establishing metabolic profile and GWAS analysis in rice, maize and tomato, several metabolic loci controlling lignin content, trigonelline level, and sugar amount have been cloned and assigned with biological functions, based on the impacts on plant height^45^, grain width^48^ and fruit size^49^. In this study, we adopted the same strategy to determine the genetic connection between cell-wall biosynthesis and grain yield-related traits. Correlation coefficient analysis revealed that all the observed cell-wall composition showed varied degrees of correlation with grain yield-related traits. The cellulose and galactose content was correlated with PH and HD and with TGW and GWD, respectively. Meanwhile, XA ratio was associated with PH and HD, as well as with GL and TGW. The combined QTL analysis revealed several overlapped loci for both sets of traits, providing genetic clues to the association. The inferred genetic links were strengthened by exploring the correlated phenotypes between cell-wall composition (*qCel6*, *qXA7-2* and *qGal8*) and plant height (*qPH6-1*), heading date (*qHD7*) or grain weight (*qTGW8*) in the corresponding CSSLs. Fine-mapping assay narrowed *qCel6* down together with *HD3a*, *HD3b* and *HD17*, QTLs for heading date^55,56^, within ~800 kb region. Investigation of allelic variations in these genes found seven SNPs in *HD17* between 9311 and NP, which introduce five nonsynonymous mutations, indicating *HD17* as a candidate of *qCel6*. Further studies, such as QTL cloning and transformation verification, are required to ensure the authentic effect of *HD17* on cellulose content. Our study thus provides genetic connection for both sets of traits, which may benefit for understanding the molecular basis of grain yield control, and for assigning biological functions to cell wall synthesizing genes.

## Methods

### Plant materials

To develop the CSSLs, the previously reported 57 CSSLs^50^ generated by using NP as recipient and 9311 as donor were subjected to a series of backcross with NP and combined with self-crosses. In total, 351 CSSLs were obtained. After genotyping with molecular markers, 138 core lines were chosen and subjected to re-sequencing. Based on the resequencing data, 125 CSSLs that cover 95.6% the 9311 genome were chosen for further phenotyping and QTL mapping.

### Field planting

The two parents and 125 lines of CSSLs were grown in the experimental fields located at the campus of Yangzhou University and at the Jiudian County of Yangzhou, Jiangsu Province, China, respectively. N62 and the related backcross population were grown in the experimental fields located at the Jiudian County of Yangzhou, Jiangsu Province and at the Lingshui County of Sanya, Hainan Province, China, respectively. Eighty plants (8 × 10) of two parents and each CSSL were planted in the plots at 25 cm interval in the horizontal direction and at 15 cm interval in the longitudinal direction.

### Measurement of grain yield-related traits and data analysis

Heading date of 125 lines and two parents were evaluated by counting the dates from sowing to flowering. Heading date of two parents and each line was recorded when more than 50% individual plants started heading. After maturation, ten representative plants of two parents and each line were subjected to measure the height from ground to the tallest panicle. One month later after seed harvest, seeds derived from 16 individual plants of two parents and each line were pooled and subjected to measure grain size and grain weight. Grain length, grain width, and grain length to width ratio were measured by a camera and analyzed with Smart Grain equipped (Version 1.1). Thousand-grain weight was measured by weighing 200 seeds from two parents and each line for three biological replicates. The data was analyzed using an independent sample *t* test program (SPSS version 15.0). Correlation coefficient between different traits was analyzed by SPSS software (version 15.0).

### Scanning electronic microscopy

The outer surface of glumes from mature seeds was sprayed with gold particles and observed with a scanning electron microscope (S-3000N, Hitachi) at an acceleration voltage of 10 kV. At least 500 cells from 12 individual seeds of two parents were measured and quantified.

### Cell-wall composition analysis

The cell-wall composition was analyzed as described previously^64^. In brief, the mature 2^nd^ internodes of two parents and CSSLs were ground into powder and treated with 70% ethanol and a mixture of chloroform and methanol (1:1 v/v) twice to prepare alcohol insoluble residues (AIRs). The de-starched AIRs were obtained by treatment with pullulanase M1 (Megazyme) and α-amylase (Sigma) in 0.1 M sodium acetate buffer (pH 5.0) for at least 24 h. After hydrolyzed in 2 M trifluoroacetic acid (TFA) at 121 °C for 90 min, the released sugars were reduced with sodium borohydride (10 mg mL^−1^ in 1 M ammonium hydroxide). Samples were analyzed by an Agilent 7890 series GC equipped with a 5975 C MS detector (Agilent). Meanwhile, crystalline cellulose content was quantified by anthrone assay^65^ using the remains after TFA treatment. The data were presented as the mean of four biological repeats.

### Molecular markers

Molecular markers developed for reconstruction of CSSLs in this study were classified into two types. Rice microsatellite (RM) markers were selected from rice microsatellite maps. The sequence and location information of these markers are available from the Gramene website (http://www.gramene.org). Sequence tagged site (STS) markers were developed by using PRIMER PREMIER 5.0 software according to the publicly available rice genome sequence (http://www.ncbi.nlm.nih.gov/). The polymorphic markers that were validated in the two parents were used for marker assistant selection.

### Resequencing of the CSSLs

Five microgram of genomic DNA from each sample was randomly fragmented by sonication and size-fractionated using electrophoresis. DNA fragments of ~500 bp were then purified. Adapter ligation and DNA clustering were performed according to the procedure from manufacturer. The fragmental samples were sequenced by Illumina HiSeq. 2000 according to the manufacturer instructions (lllumina, San Diego, California, USA). Read sequences were aligned with the reference pseudomolecules (MSU Rice Genome Annotation Project, http://rice.plantbiology.msu.edu/) and applied for SNP detection.

### QTL mapping

ICIMapping 4.0 software was used to detected the QTLs by the aid of constructing Bin-map in which the genotype of 9311 and NP was assigned as 2 and 0, respectively^66^. The QTL loci were determined by a likelihood ratio test based on single point analysis. QTLs with logarithm of the odds ratio (LOD) larger than 2.5 were considered as real QTLs. The allelic effect was calculated as the mean effect of replacing NP alleles by 9311 alleles at the QTL. The trait value and QTL located chromosome number were indicated. Mapchart (version 2.2) was applied to locate the QTLs on the Bin-map.

### Construction of Bin-map

To locate the donor segments in the CSSLs for QTL mapping, Bin-map was constructed based on the above SNP genotyping analyses. The detected SNPs were placed onto the chromosomes according to the physical location. The recombination breakpoints and donor segments were determined using a sliding window approach according to the method described^50^. Finally, 357 Bins were generated. The physical length of bins ranges from 0.01 to 8.92 Mb.

## Electronic supplementary material

Supplemental Figures
