# Supplementary material for: Genetic connection between cell-wall composition and grain yield via parallel QTL analysis in indica and japonica subspecies
Source: Sci Rep. 2017 Oct 2;7:12561. doi: 10.1038/s41598-017-12903-5 (PMC5624937; doi:10.1038/s41598-017-12903-5)
Supplement: Supplementary file 1 — Supplemental Figures [file 41598_2017_12903_MOESM1_ESM.pdf]

**Genetic connection between cell-wall composition and grain yield via parallel QTL analysis in *indica* and *japonica* subspecies**

Zuopeng Xu[1†], Shance Li[2,4†], Changquan Zhang[1, 3†], Baocai Zhang[2], Kongzhi Zhu[1], Yihua Zhou[2, 4]*and Qiaoquan Liu[1, *]

[1]Jiangsu Key Laboratory of Crop Genetics and Physiology/Key Laboratory of the Ministry of Education for Plant Functional Genomics, College of Agriculture, Yangzhou University, Yangzhou 225009, China

[2]State Key Laboratory of Plant Genomics, Institute of Genetics and Developmental Biology, Chinese Academy of Sciences, Beijing 100101, China

[3]Co-Innovation Center for Modern Production Technology of Grain Crops of Jiangsu Province/Joint International Research Laboratory of Agriculture and Agri-Product Safety of the Ministry of Education, Yangzhou 225009, China

[4]University of Chinese Academy of Sciences, Beijing 100049, China

[†]These authors contributed equally to this work.

*E-mail: yhzhou@genetics.ac.cn and qqliu@yzu.edu.cn

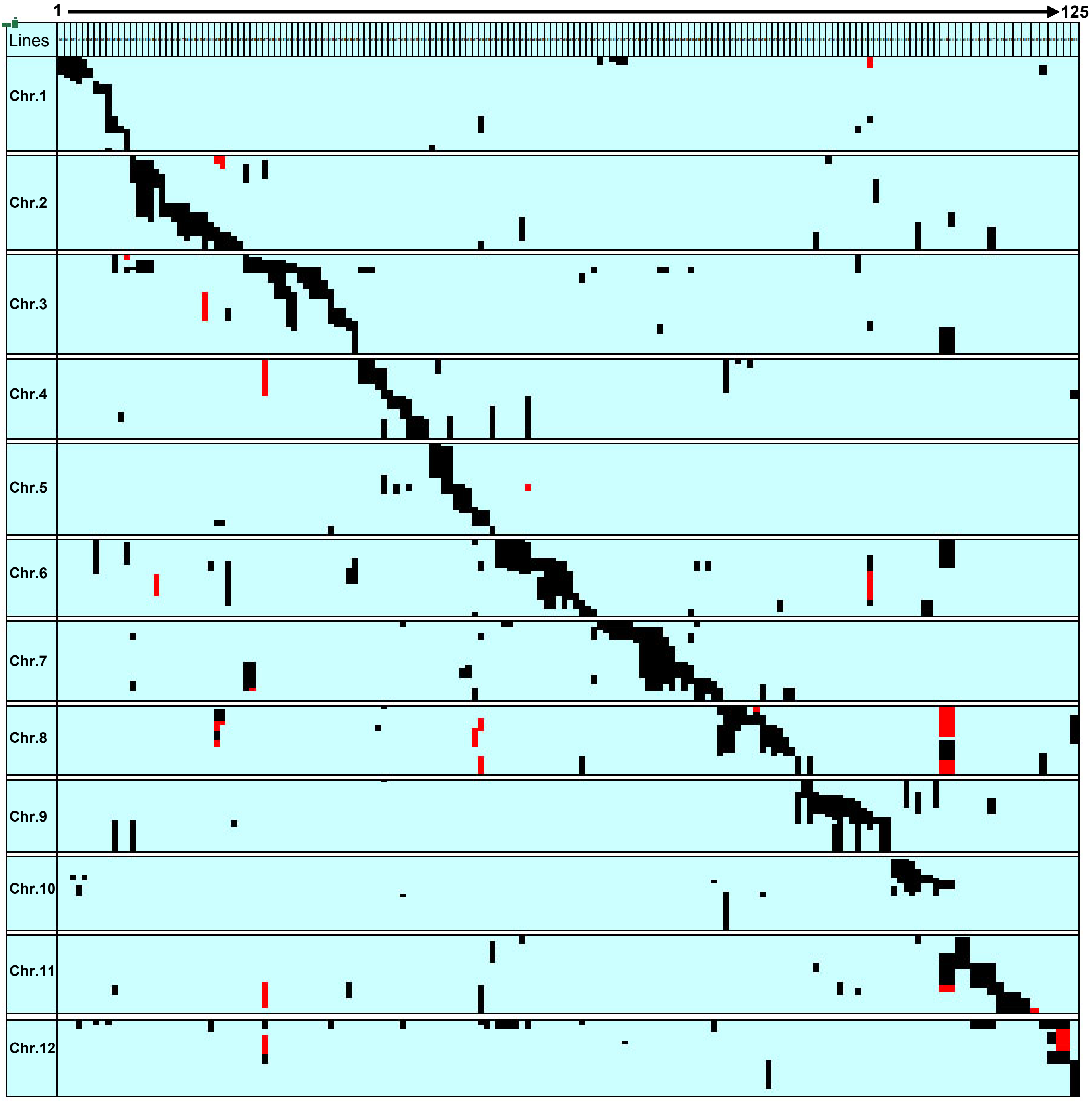


**Supplemental Figure 1. Construction of the physical map of 125 CSSLs based on the whole-genome resequencing data.** The black bars indicate the substituted segments from 9311. The light blue regions indicate NP background and the red bars indicate the 9311-NP heterozygous segments.

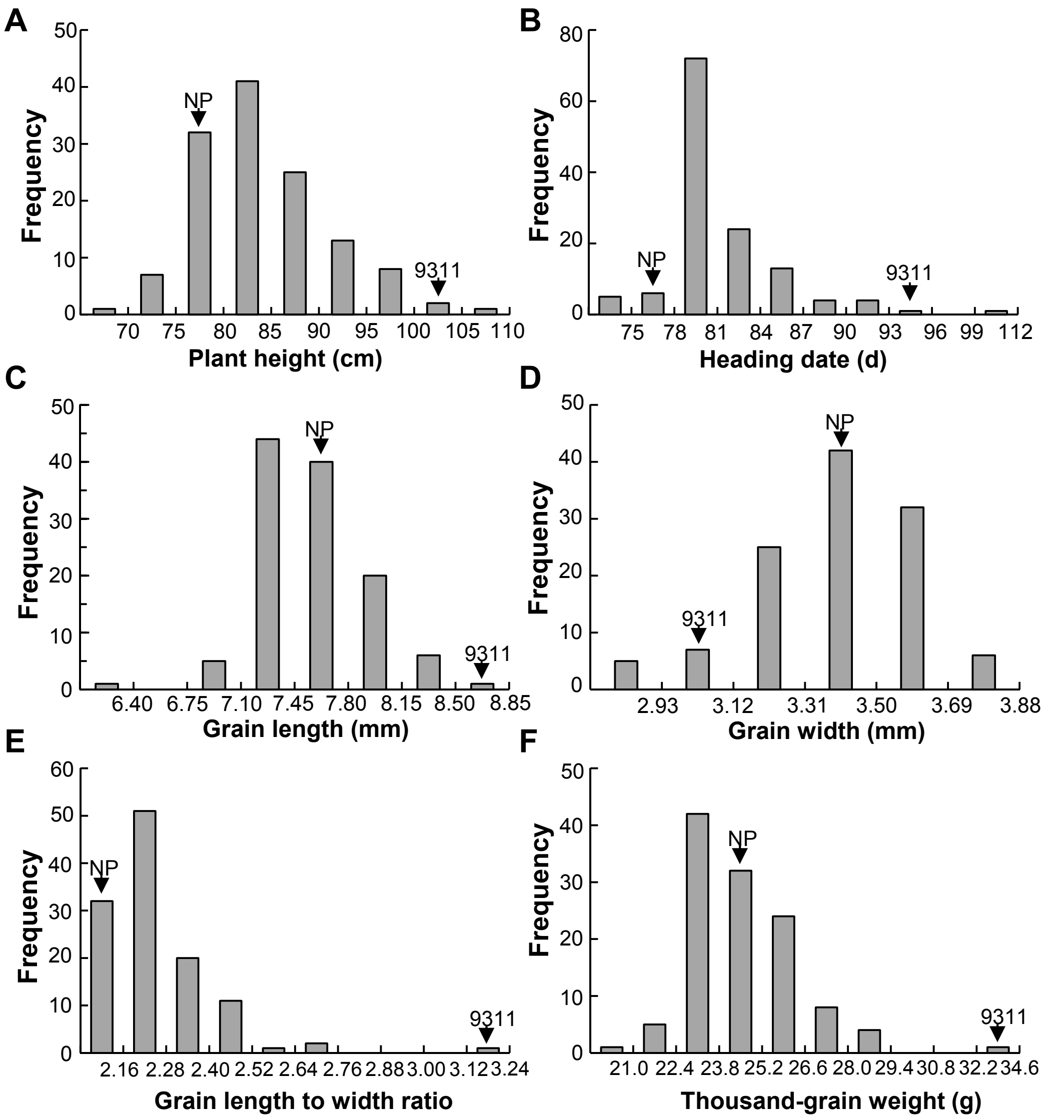


**Supplemental Figure 2. Frequency distribution of plant height, heading date, and grain size/weight in the CSSLs and parents.** (**A**) Plant height. (**B**) Heading date. (**C**) Grain length. (**D**) Grain width. **(E)** Grain length to width ratio. (**F**) Thousand-grain weight. Arrows indicate recipient parent NP and donor parent 9311.

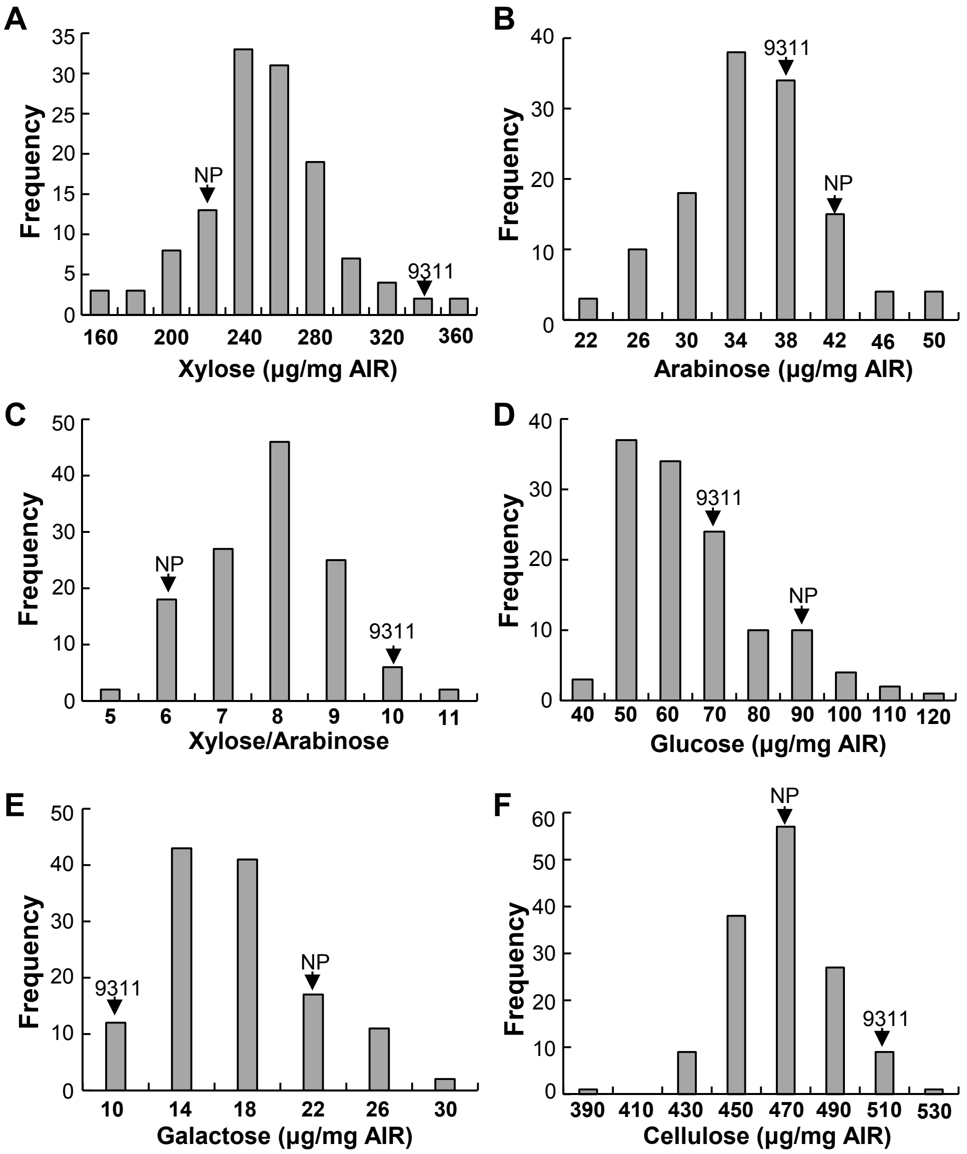


**Supplemental Figure 3. Frequency distribution of cell wall composition in the internodes of CSSLs and parents**. (**A**) The xylose content. (**B**) The arabinose content. (**C**) Xylose/Arabinose ratio. (**D**) The glucose content. (**E**) The galactose content. (**F**) The cellulose content. Arrows indicate recipient parent NP and donor parent 9311.

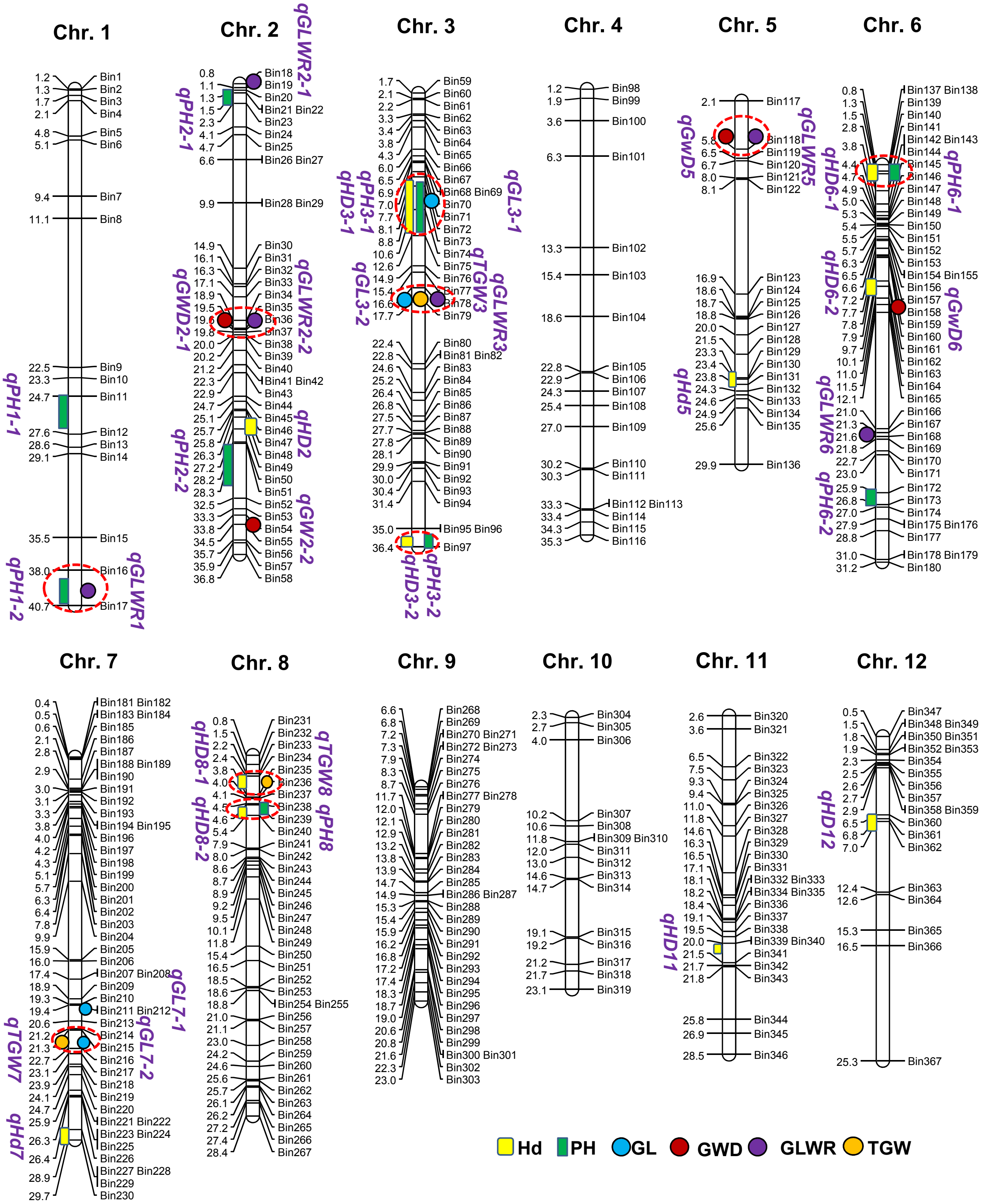


**Supplemental Figure 4. Chromosomal locations of the QTLs for grain yield-related traits.** Different symbols indicate the positions where the corresponding QTLs are located. The red dash lines circle the co-localized QTLs. The numbers at the left side of chromosomes indicate the physical locations (Mb).

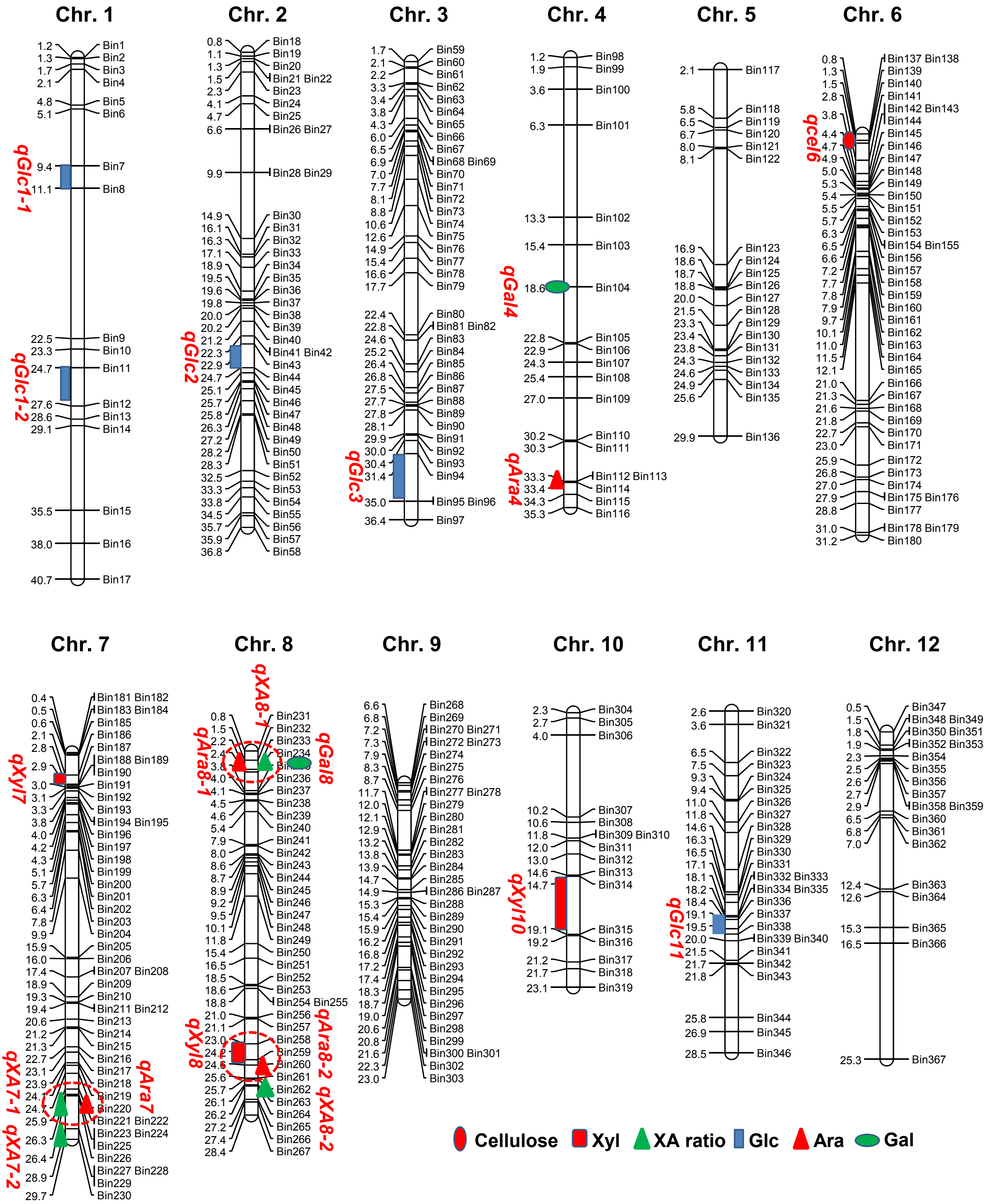


**Supplemental Figure 5. Chromosomal locations of the QTLs for cell-wall composition**. Different symbols indicate the positions where the corresponding QTLs are located. The red dash lines circle the overlapped QTLs. The numbers at the left side of chromosomes indicate the physical locations (Mb).
